# Randomized clinical trial of vaginal microbiota in menopausal genitourinary syndrome using radiofrequency and topical estriol

**DOI:** 10.61622/rbgo/2025rbgo85

**Published:** 2025-10-21

**Authors:** Ana Ximena Zunino, Priscila de Almeida Torre, Susana Cristina Aidé Viviani, Isabel Cristina Chulvis do Val Guimarães, Caroline Alves de Oliveira Martins, Douglas Guedes Ferreira, Luiza Oliveira Ribeiro, Faustino Ramón Pérez-López

**Affiliations:** 1 Universidade Federal Fluminense Niteroi RJ Brazil Universidade Federal Fluminense, Niteroi, RJ, Brazil.; 2 Universidade De Medicina da Zaragoza Zaragoza Spain Universidade De Medicina da Zaragoza, Zaragoza, Spain.

**Keywords:** Atrophic vaginitis, Estrogen replacement therapy, Radiofrequency ablation

## Abstract

**Objective:**

To analyze the vaginal microbiota before and after the treatment with vaginal microablative fractional radiofrequency (FRAXX) and compare it with topical estriol treatment in women with GMS.

**Methods:**

Pilot clinical trial, double-blind, randomized, and placebo-controlled. Thirty women diagnosed with SGM were evaluated regarding the vaginal microbiota before and one month after the end of the treatment protocol, through culture, bacterioscopy by Gram and pHmetry. Then, they were randomized into two groups: one with application of topical estriol for 21 days of attack and then 3 times a week until completing 3 months, together with placebo radiofrequency (RF) pulses, and the other group, with monthly radiofrequency pulses for 3 months, together with placebo vaginal cream.

**Results:**

The average age of participants was 56.33 ± 7.10 (minimum 42 and maximum 69 years of age), average age at onset of menopause 44.70 ± 5.74. In the estriol group, the participants showed no significant difference after treatment in relation to the number of basal and parabasal cells (p = 1.000), type of vaginal microbiota (p = 0.544), presence (or absence) of *Lactobacillus* (p = 1.000), presence (or absence) of *Coccobacillus* (p = 1.000), presence (or absence) of grouped or chained *Cocci* (p = 1.000), except for the presence of *Candida* (p = 0.025) and decrease in vaginal hydrogen potential (pH) (p = 0.006). In the group treated with FRAXX, it was observed that the participants showed no significant difference after treatment regarding the number of basal and parabasal cells (p = 0.500), type of vaginal microbiota (p = 0.637), presence (or absence) of *Lactobacillus* (p = 1.000), presence (or absence) of *Cocobacilli* (p = 1.000), presence (or absence) of grouped or chained *Cocci* (p = 1.000) and presence (or absence) of *Candida* (p = 1.000), except for pH variation (p = 0.037). Comparing the groups, women treated with FRAXX had a higher proportion of *Lactobacillus* (type I) than women treated with estriol (66.7% and 26.7%, respectively; p = 0.057). On the other hand, the proportional presence of other bacteria, but with a predominance of *Lactobacillus* (type II a) was higher in the group treated with estriol, when compared to the FRAXX group (46.6% and 6.7%, respectively; p = 0.057). And the application of estriol significantly increased the *Candida* concentration when compared to FRAXX (p = 0.042).

**Conclusion:**

There was improvement in the parameters analyzed regarding the vaginal microbiota in the intervention with FRAXX with a higher proportion of *Lactobacillus* and a decrease in pH. And there was no superiority of FRAXX in relation to the use of topical estriol.

## Introduction

Genitourinary syndrome of menopause (GSM) is defined as a collection of symptoms and signs associated with decreased levels of estrogen and other sex steroids.^(1)^ The changes caused by reduced estradiol secretion can lead to genital symptoms including dryness, burning, and irritation. It can also alter epithelial morphology, such as thinning of the vaginal epithelial surface, reduced fluid secretion, and decreased Lactobacillus levels, leading to increased vaginal hydrogen potential (pH) and favoring pathogen infections such as Escherichia coli and Mobiluncus, leading to a predisposition to urinary tract infection.^(1)^

GSM affects up to 84% of women, starting from the climacteric period. However, more than 70% of symptomatic women do not complain, recognize, or report these symptoms to their healthcare providers.^(1-3)^ The diagnosis of GSM is clinical and can be confirmed through various methods. These include testing vaginal pH and performing a vaginal smear to assess the Frost index, or Vaginal Health Index Score (VHIS) can be used.^(4)^

Unlike other menopausal symptoms that tend to improve over time, GSM is a chronic condition with symptoms that worsen when not treated.^(5-8)^ The treatment of GSM can be conducted with hormonal therapy or non-hormonal approaches such as minimally invasive energy-based therapies (laser and radiofrequency therapies). With the treatment of genitourinary signs and symptoms achieved by radiofrequency, there is an expectation of greater adherence from women due to its more convenient dosing regimen. However, the high cost remains a limiting factor. Additionally, this therapy is safer for women who have contraindications to estrogen therapy.^(8-11)^ Studies have shown positive outcomes and good acceptance of these methods in 84% of women undergoing treatment, with improvement in the VHIS, indicating enhanced vaginal health.^(10,11)^ This index evaluates parameters such as elasticity, pH, fluid volume, integrity, and hydration of the vaginal wall. Scores range from 5 to 25, with vulvovaginal atrophy (VVA) diagnosed when the resulting score is less than 15.^(12)^

Several therapeutic strategies have been proposed for the treatment. It is recognized that vaginal estrogen can improve symptoms. However, non-hormonal approaches can be useful in specific cases where hormones are not recommended, such as in patients with thromboembolism risks or in breast cancer survivors.^(2-6)^ Recently, physical methods such as light amplification by stimulated emission of radiation (laser) or radiofrequency in their non-ablative, ablative, and micro ablative forms have been used to promote collagenesis, elastogenesis, and neovascularization in the vaginal mucosa.^(7)^ Currently, some studies have shown positive results with the application of laser or radiofrequency in improving vaginal mucosa, becoming a reasonable therapeutic option for women who have contraindications or do not wish to use hormonal therapies.^(7-9)^ The vaginal microbiota of postmenopausal women, due to estrogen reduction, results in depletion of lactobacilli, thinning of the vaginal mucosa, decreased glycogen production and consequent reduction in the production of lactic acid with an increase in vaginal pH which, in turn, increases colonization by harmful microorganisms such as Escherichia coli, Candida Albicans, Enterobacter, Gardnerella, favoring infections.^(10,11)^ This study aims to analyze the vaginal microbiota before and after treatment using microablative fractional radiofrequency (MFR) on the vaginal mucosa of women with GSM. It also seeks to compare the vaginal microbiota following MFR treatment with topical estriol therapy.

## Methods

**Figure 1:**
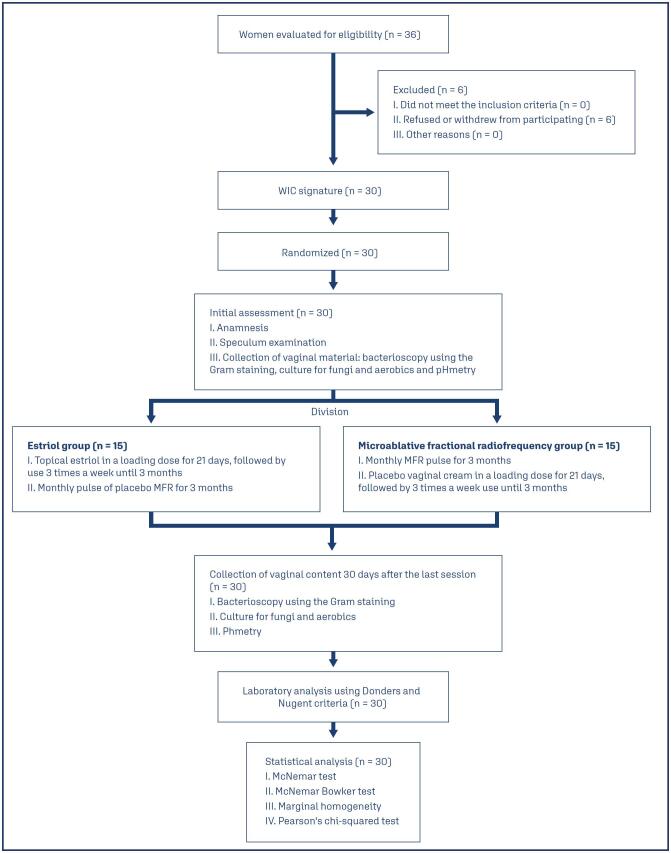
Flowchart of the study

The pilot study is a randomized, double-blind, placebo-controlled clinical trial conducted at the Gynecology Service of the Antonio Pedro University Hospital. The study took place from November 2021 to December 2022, initially with 36 participants, although six women withdrew from the project due to scheduling conflicts. The flowchart (Figure 1) describes the sequence of actions taken to manage study participants. The study was registered in the Brazilian Clinical Trials Registry with the number 14,545.

WIC – Written informed consent; MFR – Microablative fractional radiofrequency

The sample calculation was based on the research by Kingsberg et al.^(12)^ and the pilot study by Kamilos et al.^(13)^ who compared the use of estriol and radiofrequency which brought them closer to conditions to be analyzed, so that, after measuring the effect size of 0.05, the number of 64 participants was reached. However, with the COVID-19 pandemic, there was a reduction in the flow of women referred to our hospital. Therefore, it was not possible to obtain the expected number of participants.

The participants signed a Written Informed Consent (WIC) and were randomly allocated into two groups, as follows: (i) the MFR group: women who were treated using this technique and with placebo topical cream; and (ii) the estriol group including women treated with topical estrogen and with a placebo sham radiofrequency pulse. Randomization was carried out using the Random Sequence Generator portal (https://www.random.org/sequences/). Both groups had the same treatment period, totalizing three radiofrequency sessions with a 30-day interval between each session, and topical estrogen three times a week for three months, after 21 days of continuous use. Before the procedure, a speculum examination and VHIS application were performed. It evaluates parameters such as elasticity, pH, fluid volume, integrity, and hydration of the vaginal wall. After the procedure, participants were instructed on the use of vaginal cream, beginning one week after the first radiofrequency application, for 21 days continuously in the first month, and then maintaining its use three times per week.

Women with genitourinary symptoms, postmenopausal serum hormone levels (follicle-stimulating hormone greater than 40 mlU/mL; estradiol less than 25 pg/mL), bilateral surgical oophorectomy for two years or more,^(12)^ not using any form of oral or topical hormonal therapy in the past three months, and with negative oncotic vaginal cytology (pap smear) results for neoplasia as recommended by the Brazilian Guidelines for Cervical Cancer Screening from the Ministry of Health.^(14)^ Women were excluded if they had human immunodeficiency virus (HIV) infection, cancer with a history of previous pelvic radiotherapy, myeloproliferative diseases, malnutrition, decompensated diabetes mellitus (glycated hemoglobin greater than 6.5%),^(15)^ history of breast cancer, estrogen-sensitive tumors, or thromboembolism. In addition, women using immunosuppressive substances were also excluded.

The participants were instructed to abstain from sexual intercourse and refrain from vaginal douching to a minimum of four days before the procedures. A thorough medical history, physical examination, collection of vaginal content for cytology and for culture to analyze vaginal microbiota and pH monitoring (as part of VHIS evaluation). For vaginal cytology, the material was collected with an Ayre spatula from the middle third of the lateral wall of the vagina, spread on a fresh slide. And placed in a dry jar intended for bacterioscopy using Gram staining for analysis of anaerobes following the Nugent et al. ^(15)^ and the Donders et al.^(16)^ criteria. They were conducted at two time points: before the start of treatment (T0) and 30 days after the completion of the third and final radiofrequency session (T4).

For the culture, two samples were taken in two swabs, also from the middle third of the lateral wall of the vagina. One was used as culture in a specific medium for fungi, and the other for research into common pathogens, Trichomonas vaginalis, Staphylococcus aureus, Streptococcus agalactiae, and Klebsiella pneumoniae, which were analyzed under aseptic conditions and seeded on solid media. The sample for aerobic bacteria was seeded onto solid media in three agar plates: chocolate, blood, and MacConkey, in that specific order, using the streak plate technique. In this technique, the bacterial suspension, which is the liquid containing the microorganism, is spread randomly across the plate until all the material is used. This ensures even distribution of growth, ensuring thorough subsequent analysis.^(17)^ The plates were then incubated in a bacteriological incubator at 37°C for up to 48 hours. The sample for fungi was seeded onto solid media in three agar tubes: Mycosel, Brain Heart Infusion agar with chloramphenicol, and Sabouraud agar with chloramphenicol, in that specific order, also using the streak plate technique. The tubes were then incubated in a bacteriological incubator at 37°C for 30 days. The analyses were carried out by the Clinical Pathology Department of the Antonio Pedro University Hospital. For pHmetry, the MQuant R brand, a range from 0 to 14 was used. The sensitive colorimetric paper strip was placed in intimate contact with the lateral vaginal wall using Cheron forceps. The color change on the strip, according to the manufacturer's reference colors, indicated the pH range of the area touched by it before the start of treatment, with parameters recorded according to the VHIS.

The collection of the material, application of the VHIS, and analysis of all microbiology were performed by a single examiner blinded to the group to which the participant belonged.

The participants underwent a vaginal examination using a disposable Collin vaginal speculum, after applying a topical anesthetic spray of 10% Xylestesin^®^, from Cristália, to the vaginal vestibule and lower third of the vagina. After waiting for 3 minutes for the anesthetic to take effect, any excess was removed with distilled water using gauze and Cheron forceps. Subsequently, vaginal asepsis was performed using 0.2% aqueous chlorhexidine from Riohex^®^. The procedure utilized the Wavetronic 6000–ACEL 1462 Touch^®^ device, set to mega pulse low soft treatment parameters, connected to a Linly^®^ vaginal fractional electrode with 64 micro-needles measuring 200 microns in diameter and spaced 1 mm apart in a grid of eight columns and eight rows. Activated by a pedal, the 64 needles were energized randomly, with energy distributed in randomized columns in a pre-sequence so that two consecutive needles were not energized simultaneously. This randomized needle mechanism prevents coagulation between points and preserves adjacent tissues during vaporization, leading to neocollagenesis, neoelastogenesis, and neovascularization through fibroelastic stimulation. Each point of the pen performs 64 micro-ablations on the mucosa. The electrode remained parallel and lightly in contact with the vaginal walls with each pedal trigger. The average duration of each procedure was 15 to 20 minutes, respecting the individual needs of each participant. All participants were instructed to avoid sexual intercourse for seven days following the procedure.

The radiofrequency device was activated via the conductor cable to ensure energy emission in the active group. In the placebo group, the cable was disconnected, but in both situations, there was noise and a sensation of energy emission, which participants in the placebo group were unaware of. Another examiner who was not blinded performed the procedure.

The vaginal cream provided to both groups was compounded at the same pharmacy, Aquarius, National Registry of Legal Entities: 11.743.485/0001-20. It consisted of a base cream with 1 mg of estriol per gram of cream for the active group. The creams were prepared with identical labels and packaging, separated into containers labeled "with" or "without" active ingredients. They were distributed to participants according to their group assignment, with each dose containing 1 gram of cream administered using vaginal applicators with measurements. Participants were unaware of which group they belonged to. The distribution of creams was carried out by the same non-blinded examiner who performed the radiofrequency procedure.

For the evaluation of the leukocyte/epithelial cell ratio, the slide was examined under 400x magnification (objective lens of 40x and ocular lens of 10x). The proportion of leukocytes to epithelial cells was observed per field. ^(15)^ The presence of granulations in the leukocytes, indicative of cytotoxic leukocytes, was observed. The number of "normal" and cytotoxic leukocytes was then compared and documented.^(14)^ The evaluation of the count of deep basal and parabasal cells was conducted by examining the slide under 400x magnification (using a 40x objective lens and a 10x ocular lens). The presence of basal and parabasal cells per field was observed and counted.^(15)^

For the analysis of the microbiota type, the reading was performed at 1000 x magnification, as described by Donders et al. criteria.^(16)^

- I – Presence of only lactobacilli.
- IIa – Presence of other bacteria, but a predominance of lactobacilli.
- IIb – Observation of lactobacilli, but predominance of other bacteria.
- III – Absence of lactobacilli, presence only of other bacteria.

The characteristics of the microbiota were analyzed by reading at 1000x magnification and observed according to Nugent's criteria: 15

Lactobacilli – Gram + long rods.

Coccobacilli/Bacilli – coccobacilli and/or Gram – bacilli.

Cocci – Gram + cocci grouped or in chains.

The Statistical Package for the Social Sciences software, version 20.0 from the International Business Machines Corporation, was used for the statistical analysis. The statistical analysis began with a description of the data for the quantitative variable age, which was described using the mean ± standard deviation when it had a normal distribution or median (first quartile [Q1]: 25% of participants have a value less than or equal to Q1 and 75% of participants have a value above); and third quartile [Q3]: 75% of participants have a value less than or equal to Q3 and 25% of participants have a value above). When it did not have a normal distribution, the variable was checked using the Shapiro-Wilk test. Categorical variables were described using absolute frequencies and percentages.

The McNemar, McNemar-Bowker, and marginal homogeneity tests were used to compare the before and after categorical variables for the same group. The McNemar test was used when the matrix had a dimension of 2 × 2, the McNemar-Bowker test when there was a square matrix with a dimension above 2 × 2, and the marginal homogeneity test when there was no square matrix. Categorical variables were compared between the two groups using Pearson's asymptotic chi-square test (20% of the expected value less than 5 and 80% of the expected value greater than 5) and Pearson's exact chi-square test (more than 20% of the expected value less than 5). When there was statistical significance in the asymptotic and exact Pearson chi-square tests (greater than 2 × 2), the standardized adjusted residuals analysis was carried out to show where the difference lies (since it is not a 2 × 2 table), (residual greater than or equal to +1.96) the cell where the percentage was higher (residual less than or equal to −1.96) the cell where the percentage was lower. There is no significant difference in the other residues.

The statistical significance level adopted was 0.05, and the study comprised 30 women.

The odds ratio (OR) was used to assess the chance of an event due to exposure (Estriol or MFR). To do this, the number of observations of the presence of the outcome in the MFR group was divided by the absence of the outcome in the Estriol group (numerator), and, subsequently, the same was done in the MFR group (denominator). Finally, the numerator was divided by the denominator. Thus, when there is OR > 1, it is understood that the chance of the event is greater in the Estriol group than in MFR, whereas when the OR < 1, it means that the chance of the event is lower in the Estriol group than in MFR and when the OR = 1, it means that the chances of the event are equal in the groups.

Participant selection began after the project was duly approved by the Research Ethics Committee of the School of Medicine at Fluminense Federal University (Certificate of Presentation of Ethical Appreciation number 49385021.6.0000.5243) and registered on the Clinical Trials Registry platform number 14545, technical opinion 5.705.213 Patients received informed consent after an explanation of the clinical and research protocol.

## Results

The study included a total of 30 women. Using the statistical software G*Power 3.1.9.7 and assuming a proportion of 0.33 for no change in microbiota, a test power of 62% was observed. The mean age was 56.00 ± 7.62 years, and the mean age at menopause was 44.70 ± 5.74 years. The variable age followed a normal distribution, described by mean ± standard deviation, as confirmed by the Shapiro-Wilk test. The duration of menopause had a median of 10.00 years, with Q1, Q3 (4.00; 16.00) (Table 1).

**Table 1:** Study population

Variables	
Age (mean±SD)	56.00±7.62
Age at menopause	44.70±5.74
Time since menopause	10.00(4.00;16.00)

When the data were evaluated by comparing variables within each experimental group, it was observed that in the estriol-treated group, participants did not show significant differences after treatment regarding the number of basal and parabasal cells (Table 2) (p = 1.000), type of vaginal microbiota (Table 2) (p = 0.544), presence (or absence) of Lactobacillus (Table 2) (p = 1.000), presence (or absence) of Coccobacilli (Table 2; p = 1.000), and presence (or absence) of clustered or chain-forming cocci (Table 2) (p = 1.000). Women treated with estriol showed a higher proportion of Candida presence after treatment (Table 2) (p = 0.025). Additionally, these women had a greater proportion of pH variation after treatment (Table 2) (p = 0.006). It was observed that in 9 women, there was a decrease in pH value. Four women (26.7%) with a pH of 6.1 before treatment had their pH decrease to 4.7–5.0 afterward. Two women (13.3%) with pH ≥ 6.1 before treatment moved to pH between 5.1–5.5. Three women (20%) shifted from the 5.1–5.5 category to 4.7–5.0. The remaining 6 participants (40%) did not show any pH alteration. Among the women who did not show pH changes, 2 (13.3%) remained in the 5.1–5.5 category, and 4 (26.7%) remained in the 4.7-5.0 category.

**Table 2:** Comparison of variables before and after treatment for the estriol group

Amount of basal and parabasal cells		After				p-value
		≤10 (n=7)		>10 (n=8)		
Before	≤10 (n=6)	6(40.0)		0(0.0)		1.000a
	>10 (n=9)	1(6.7)		8(53.3)		
	Type of microbiota	I (n=4)	IIa (n=7)	IIb (n=3)	III (n=1)	
Before	I – Presence of only lactobacilli (n=4)	1(6.7)	2(13.3)	1(6.7)	0(0.0)	0.544b
	IIa – Presence of other bacteria, but predominance of lactobacilli (n=4)	2(13.3)	2(13.3)	0(0.0)	0(0.0)	
	IIb – Observation of lactobacilli, but predominance of other bacteria (n=4)	0(0.0)	2(13.3)	1(6.7)	1(6.7)	
	III – Absence of lactobacilli, presence of only other bacteria (n=3)	1(6.7)	1(6.7)	1(6.7)	0(0.0)	
	Presence (or absence) of Lactobacillus	Absent or rare (n=5)		Moderate or numerous (n=10)		
Before	Absent or rare (n=6)	3(20.0)		3(20.0)		1.000a
	Moderate or numerous (n=9)	2(13.3)		7(46.7)		
	Presence (or absence) of Coccobacillus or bacilli	Absent or rare (n=12)		Moderate or Numerous (n=3)		
Before	Absent or rare (n=12)	10(66.7)		2(13.3)		1.000a
	Moderate or numerous (n=3)	2(13.3)		1(6.7)		
	Presence (or absence) of grouped or chained Cocci	Absent or rare (n=12)		Moderate or Numerous (n=3)		
Before	Absent or rare (n=12)	10(66.7)		2(13.3)		1.000a
	Moderate or numerous (n=3)	2(13.3)		1(6.7)		
	pH	≥6.1 (n=0)	5.1 – 5.5 (n=4)	4.7 – 5.0 (n=11)		
Before	≥6.1 (n=6)	0(0.0)	2(13.3)	4(26.7)		0.006c
	5.1–5.5 (n=5)	0(0.0)	2(13.3)	3(20.0)		
	4.7–5.0 (n=4)	0(0.0)	0(0.0)	4(26.7)		
	Candida	Absent (n=10)	Present (n=5)			
Before	Absent (n=15)	10(66.7)	5(33.3)			0.025c
	Present (n=10)	0(0.0)	0(0.0)			

Data expressed as frequency (percentage); a McNemar test; b McNemar-Bowker test; c Marginal homogeneity test; pH – Hydrogen potential

When the variables were analyzed among women in the group treated with MFR, it was observed that participants did not show significant differences after treatment regarding the number of basal and parabasal cells (Table 3) (p = 0.500), type of vaginal microbiota (Table 3) (p = 0.637), presence (or absence) of Lactobacillus (Table 3) (p = 1.000), presence (or absence) of Coccobacilli (Table 3) (p = 1.000), presence (or absence) of clustered or chain-forming cocci (Table 3) (p = 1.000), and presence (or absence) of Candida (Table 3) (p = 1.000). However, women treated with MFR showed a variation in pH after treatment (Table 3) (p = 0.037). It was observed that 7 women experienced a decrease in pH value. Three women who had a pH of 6.1 before treatment saw their pH change to 4.7–5.0 afterward. One woman with pH ≥ 6.1 before treatment moved to the pH category between 5.1–5.5. One woman shifted from the 5.1–5.5 pH category to 4.7–5.0. The pH of one woman changed from 4.7–5.0 to 5.1–5.5, and the other woman changed from 5.6-6 to ≥ 6.1. The remaining participants (n=6; 40%) did not experience any pH alteration. Among those who did not show pH changes, 3 remained in the ≥ 6.1 pH category and 3 remained in the 4.7-5.0 category.

**Table 3:** Comparison of variables before and after treatment for the MFR group

Amount of basal and parabasal cells		After				p-vale
		≤10 (n=7)		>10 (n=8)		
Before	≤10 (n=5)	5(33.3)		0(0.0)		0.500a
	>10 (n=10)	2(13.3)		8(53.3)		
	Type of microbiota	I (n=10)	IIa (n=1)	IIb (n=3)	III (n=1)	
Before	I – Presence of only lactobacilli (n=6)	5(33.3)	0(0.0)	1(6.7)	0(0.0)	0.637b
	IIa – Presence of other bacteria, but predominance of lactobacilli (n=6)	3(20.0)	1(6.7)	2(13.3)	0(0.0)	
	IIb – Observation of lactobacilli, but predominance of other bacteria (n=3)	2(13.3)	0(0.0)	0(0.0)	1(6.7)	
	Presence (or absence) of Lactobacillus	Absent or rare (n=4)		Moderate or numerous (n=11)		
Before	Absent or rare (n=4)	1(6.7)		3(20.0)		1.000a
	Moderate or numerous (n=11)	3(20.0)		8(53.3)		
	Presence (or absence) of Coccobacillus or bacilli	Absent or rare (n=13)		Moderate or numerous (n=2)		
Before	Absent or rare (n=14)	13(86.6)		1(6.7)		1.000a
	Moderate or numerous (n=1)	0(0.0)		1(6.7)		
	Presence (or absence) of grouped or chained Cocci	Absent or rare (n=12)		Moderate or numerous (n= 3)		
Before	Absent or rare (n=11)	8(53.3)		3(20.0)		1.000a
	Moderate or numerous (n=4)	4(26.7)		0(0.0)		
	pH	≥6.1 (n=4)	5.6–6.0 (n=0)	5.1 – 5.5 (n=3)	4.7 – 5.0 (n=8)	
Before	≥6.1 (n=7)	3(20.0)	0(0.0)	1(6.7)	3(20.0)	0.037c
	5.6–6.0 (n=3)	1(6.7)	0(0.0)	1(6.7)	1(6.7)	
	5.1–5.5 (n=1)	0(0.0)	0(0.0)	0(0.0)	1(6.7)	
	4.7–5.0 (n=4)	0(0.0)	0(0.0)	1(6.7)	3(20.0)	
	Candida	Absent (n=14)		Present (n=1)		
Before	Absent (n=13)	13(86.6)		0(0.0)		1.000a
	Present (n=2)	1(6.7)		1(6.7)		

Data expressed as frequency (percentage); a McNemar test; b Marginal homogeneity test; c McNemar-Bowker test; MFR – Microablative fractional radiotherapy; pH – Hydrogen potential

In addition to evaluating variables within the same experimental groups (MFR or estriol), comparisons were made between these groups both before and after treatment. When women were compared based on the type of treatment received, differences were noted regarding the type of vaginal microbiota present after treatment. Women treated with MFR showed a higher proportion of Lactobacillus presence (type I) compared to those treated with estriol (Table 4) (66.7% and 26.7%, respectively; p = 0.057). Conversely, the proportion of other bacteria, with a predominance of Lactobacillus (type IIa), was higher in the estriol-treated group compared to the MFR group (Table 4; 46.6% and 6.7%, respectively; p = 0.057). Another significant difference observed when participants were compared according to the treatment received was the change in the amount of Candida. The percentage of women who showed worsening in this variable was higher in the estriol-treated group compared to the MFR group (Table 4) (33% and 0%, respectively; p = 0.042). The other variables did not differ between the experimental groups. From the assessment of the OR, highlighting only the moments after the intervention, it was noted that there was a greater chance of "Lactobacillus after: absent or rare" and "No Change basal/parabasal cells" in the Estriol group than in the MFR group (OR > 1) and greater chance of "Coccobacilli or bacilli after: Moderate or numerous" and "Candida after: Present" in the Estriol group than in the MFR group (OR < 1), in other words, there is a greater chance of "Coccobacilli or bacilli after: Absent or rare" and "Candida after: Absent" in the MFR group. The variables "Basal/parabasal cells" and "Coco grouped or in a chain" presented equal chances in the Estriol and MFR groups (OR = 1) (Table 4).

**Table 4:** Comparison of response variables studied between participants treated with estriol or MFR

		Groups			
Variables		Estriol	MFR	Odds ratio^a^	p-value
		(n=15)	(n=15)		
Basal/parabasal cells before					
	<10	6(40.0)	5(33.3)	1.33 [0.30; 5.92]	0.7052
	>10	9(60.0)	10(66.7)		
Basal/parabasal cells after					
	<10	7(46.7)	7(46.7)	1.00 [0.24; 4.29]	1.0002
	>10	8(53.3)	8(53.3)		
Type of microbiota before				-	0.2841
	I - Presence of only Lactobacillus	4(26.7)	6(40.0)		
	Ila - Presence of other	4(26.7)	6(40.0)		
bacteria, but predominance of Lactobacillus					
			
	Ilb - Observation of	4(26.7)	3(20.0)		
Lactobacillus, but predominance of other bacteria					
			
	Ill - Absence of Lactobacillus,	3(20.0)	0(0.0)		
presence of only other bacteria					
Type of microbiota after					
	I - Presence of only	4(26.7) *	10(66.7) **		
Lactobacillus					
	Ila - Presence of other	7(46.6) **	1(6.7) *		
bacteria, but predominance of Lactobacillus					
			
	IIb - Observation of	3(20.0)	3(20.0)	-	0.0571
Lactobacillus, but predominance of other bacteria					
			
	III - Absence of Lactobacillus,	1(6.7)	1(6.7)		
presence of only other bacteria					
Lactobacillus before					
	Absent or rare	6(40.0)	4(26.7)	1.83 [0.39; 8.57]	0.4392
	Moderate or numerous	9(60.0)	11(73.3)		
Lactobacillus after					
	Absent or rare	5(33.3)	4(26.7)	1.37 [0.28; 6.61]	1.0001
	Moderate or numerous	10(66.7)	11(73.3)		
Coccobacilli or bacilli before					
Absent or rare		12(80.0)	14(93.3)	0.28 [0.03; 3.12]	0.5981
Moderate or numerous		3(20.0)	1(6.7)		
Coccobacilli or bacilli after					
	Absent or rare	12(80.0)	13(86.7)	0.61 [0.09; 4.34]	1.0001
	Moderate or numerous	3(20.0)	2(13.3)		
Coco grouped or in a chain before					
	Absent or rare	9(60.0)	11(73.3)	0.54 [0.12; 2.55]	0.4392
	Moderate or numerous	6(40.0)	4(26.7)		
Coco grouped or in a chain after					
	Absent or rare	12(80.0)	12(80.0)	1.00 [0.17; 5.99]	1.0001
	Moderate or numerous	3(20.0)	3(20.0)		
Candida before					
	Absent	15(100.0)	13(86.7)	5.74	0.4831
	Present	0(0.0)	2(13.3)	[0.25;130.50]	
Candida after					
	Absent	10(66.7)	14(93.3)	0.14 [0.01; 1.41]	0.1691
	Present	5(33.3)	1(6.7)		
pH before					
	>6.1	6(40.0)	7(46.6)		
	5.6-6.0	0(0.0)	3(20.0)		
	5.1-5.5	5(33.3)	1(6.7)	-	0.1211
	4.7-5.0	4(26.7)	4(26.7)		
pH after					
	>6.1	0(0.0)	4(26.7)	-	0.1691
	5.1-5.5	4(26.7)	3(20.0)		
	4.7-5.0	11(73.3)	8(53.3)		
Change basal/parabasal cells					
	No change	14(93.3)	13(86.7)	2.15 [0.17; 26.69]	1.0001
	Improvement	1(6.7)	2(13.3)		
Microbiota change					
	Worse	4(26.7)	4(26.7)	-	0.8951
	No change	4(26.7)	6(40.0)		
	Improvement	7(46.6)	5(33.3)		
Lactobacillus Change					
	Worse	2(13.3)	3(20.0)	-	1.0001
	No change	10(66.7)	9(60.0)		
	Improvement	3(20.0)	3(20.0)		
Change Coccobacillus or bacilli					
	Worse	2(13.4)	1(6.7)	-	0.3971
	No change	11(73.3)	14(93.3)		
	Improvement	2(13.3)	0(0.0)		
Change Coconuts grouped or in chains					
				-	1.0001
	Worse	2(13.4)	3(20.0)		
	No change	8(53.3)	8(53.3)		
	Improvement	5(33.3)	4(26.7)		
Candida Change					
	Worse	5(33.3) **	0(0.0) *	-	0.0421
	No change	10(66.7)	14(93.3)		
	Improvement	0(0.0)	1(6.7)		
pH change					0.6132
	Worse	0(0.0)	2(13.3)	-	
	No change	6(40.0)	6(40.0)		
	Improvement	9(60.0)	7(46.7)		

## Discussion

The present study included 30 participants, with a mean age of 56.33 years ± 7.10 (minimum of 42 and maximum of 69 years). The average age of menopause was 44.70 ± 5.74 years, as well as in Africa, Asia, and the Middle East, which supports the findings of the present study.^(18)^

Clinical studies have demonstrated that topical application of vaginal estrogen is the gold standard treatment for GSM,^(19-24)^ improving vaginal pH and microbiota.^(25,26)^ Also in this study, in the GE group, we observed a similar improvement in pH where 9 women showed a decrease in pH values: 26.7% had pH ≥ 6.1 reduced to 4.7–5.0; 13.3% had pH ≥ 6.1 reduced to 5.1–5.5; and 20% transitioned from pH 5.1–5.5 to 4.7–5.0, with p = 0.006

In this study, an improvement in pH was observed in the MFR group, with 7 women showing a decrease in pH values: 20% of women with initial pH ≥ 6.1 decreased to 4.7–5.0; 6.7% with initial pH ≥ 6.1 decreased to 5.1–5.5; and 6.7% who had pH in the range of 5.1–5.5 decreased to 4.7–5.0. The p-value for these observations was 0.037. When both groups were compared, the pH did not differ after treatment between the Estriol group and the MFR group, with a p-value of 0.12156. Thus, MFR proves to be a plausible and non-inferior option for improving pH. Also, according to a randomized clinical trial, concluded that MRF therapy restored vaginal balance, akin to what would typically be expected with sufficient estrogen levels.^(26,30)^

Regarding vaginal microbiota, the results of the present study indicated that the group of women treated with hormone therapy (estriol group) showed a higher proportion of microbiota composed of other bacteria, but with a predominance of Lactobacillus (IIa) (46.6%) when compared to the MFR group, which had a higher proportion of women with microbiota composed exclusively of Lactobacillus (I) (66.7%). Regarding type I vaginal microbiota, characterized by a predominance of Lactobacillus, the MFR group showed a statistically significant difference compared to the estriol group (66.7% and 26.7%, respectively; p = 0.057). These results corroborate the studies by Sarmento et al.,^(2,31)^ which also found an increase in vaginal colonization by Lactobacillus after the use of MFR. The significant increase in Lactobacillus demonstrates that MFR therapy beneficially restores the vaginal mucosa for the vaginal microbiome. Therefore, it is possible to attribute to MFR similar effects as hormone therapy in the treatment of GSM concerning vaginal microbiota.

The group of women reated with estriol, in this study showed an increase in the number (33.3%) with vaginal colonization by Candida after treatment, compared to the same women (0%) before hormone use (p = 0.025). This change in vaginal microbiota was not observed in the MFR group. Based on this information, the presence of estradiol with the restoration of more acidic pH created a vaginal environment favorable to the development of Candida. A systematic review to assess the available evidence on the efficacy and safety of vaginal estrogen products for the treatment of GSM observed in the studies 34 (0.73%) cases of infection with the use of vaginal estrogen.^(30)^ This adverse effect, present in hormonal treatment, is not observed in the use of energies.

While Sarmento et al.^(31)^ demonstrated an improvement in cellular maturation, with a decrease in the percentage of parabasal cells and an increase in the rate of superficial cells, in the present study, there was no statistically significant difference in the proportions before and after treatment in either the MFR group or the estriol group, nor in the comparison between the two groups.

According to Kamilos et al.^(13,32,33)^ MFR has proven to be a promising alternative for treating GSM without the use of hormones. However, there does not appear to be, up to the present moment, robust evidence regarding the therapeutic advantages of radiofrequency for GSM compared to the gold standard, topical hormonal therapy. Our results support previous studies among Brazilian women living in Rio de Janeiro. There is a need to evaluate several aspects of this type of treatment for GSM, including cost, efficacy, long-term safety, and access to treatment.^(12,31,32,34)^

A limiting aspect encountered in this study is the fact that it was conducted during a pandemic period, a short follow-up period, and a sample size below the desired level. Future research is recommended to include a larger sample size to improve statistical relevance. Studies with long-term follow-up are also crucial to assess the effects of radiofrequency and the need for additional treatments.^(35)^ It should be noted that our group of researchers continues to monitor the women who participated in this study. Another limiting factor to mention is that this study included women with heterogeneous menopausal durations, ranging from 2 to 30 years, with a median of 10 years and Q1; Q3 (4.00; 16.00). It is believed that the duration of menopause can influence therapy outcomes due to greater loss of elastic fibers and collagen, decreased activity of estrogen receptors, and reduced glycogen production, which are naturally progressive processes in GSM.

## Conclusion

Although there was an improvement in the analyzed parameters of the vaginal microbiota with the MFR intervention, it did not show superiority over the use of topical estriol in improving the vaginal microbiota. And there is still a lack of arguments that connect our findings with the clinical impact. Therefore, there is a need to increase the number of participants and long-term follow-up to improve our findings and a possible recommendation in clinical practice.

## Data availability

: The authors did not make the data from this article available in repositories prior to submission.
